# Point-of-Care Ultrasound in the Diagnosis of Melioidosis in Laos

**DOI:** 10.4269/ajtmh.20-0069

**Published:** 2020-06-01

**Authors:** Michaëla A. M. Huson, Kerstin Kling, Somaphone Chankongsin, Khampheng Phongluxa, Valy Keoluangkhot, Paul N. Newton, David Dance, Tom Heller, Andreas Neumayr

**Affiliations:** 1 Department of Microbiology and Infectious Diseases, Erasmus Medical Centre, Rotterdam, The Netherlands;; 2 Department of Medicine, Swiss Tropical and Public Health Institute, Basel, Switzerland;; 3 University of Basel, Basel, Switzerland;; 4 Department of Infectious Disease Epidemiology, Robert Koch-Institute, Berlin, Germany;; 5 Infectious Diseases Ward, Mahosot Hospital, Vientiane, Lao People’s Democratic Republic;; 6 Lao Tropical and Public Health Institute, Ministry of Health, Vientiane, Lao People’s Democratic Republic;; 7 Lao-Oxford-Mahosot Hospital-Wellcome Trust Research Unit (LOMWRU), Microbiology Laboratory, Mahosot Hospital, Vientiane, Lao People’s Democratic Republic;; 8 Centre for Tropical Medicine and Global Health, University of Oxford, Oxford, United Kingdom;; 9 Faculty of Infectious and Tropical Diseases, London School of Hygiene and Tropical Medicine, London, United Kingdom;; 10 Lighthouse Clinics, Lilongwe, Malawi

## Abstract

Melioidosis is endemic in many rural areas in Southeast Asia where facilities for culture and identification of *Burkholderia pseudomallei* are often limited. We performed a prospective observational study in patients presenting with fever to Mahosot Hospital, the primary referral hospital in Laos, to establish whether the detection of abscesses on ultrasound could support a presumptive diagnosis of melioidosis. All patients underwent ultrasound examination to detect abscesses in the liver, spleen, prostate, or, if indicated, subcutaneous tissue. We enrolled 153 patients, including 18 patients with melioidosis. Of these, 11 (61%) had an abscess at one or more sites, including five (28%) with splenic and/or liver abscesses. Absence of abscesses cannot rule out melioidosis, but the positive predictive value of abscesses for melioidosis was high at 93% (88–96%). Therefore, in endemic areas, the presence of abscesses in febrile patients should prompt empiric antibiotic therapy for melioidosis even in the absence of culture confirmation.

Over the past two decades, ultrasound has evolved as a noninvasive, reproducible, low-cost diagnostic method in many disciplines of clinical medicine. In addition, the development of low-priced transportable ultrasound machines has created opportunities in resource-constrained settings where other diagnostic imaging methods remain largely unavailable. Growing availability of portable ultrasound machines has also led to the establishment and implementation of numerous “point-of-care ultrasound” (POCUS) protocols in various medical disciplines, including tropical medicine.^1–3^ For example, in settings with a high prevalence of HIV and tuberculosis, the Focused Assessment with Sonography for HIV/TB (FASH) protocol is widely used to assess for signs of extrapulmonary tuberculosis.^1,4^ Melioidosis is an infectious disease caused by the Gram-negative bacterium *Burkholderia pseudomallei*. The disease is primarily reported in Southeast Asia and Northern Australia, but increasingly recognized to also occur in sub-Saharan Africa and South America.^5^ In parts of Southeast Asia, including Laos,^6^ melioidosis is an important differential diagnosis in patients with fever and/or sepsis. Risk factors for infection include diabetes, excessive alcohol use, chronic renal disease, chronic lung disease, thalassemia, malignancy, and other non-HIV–related immune suppression. Because the clinical presentation of melioidosis is often unspecific and the laboratory capacity for microbiological confirmation is frequently limited in endemic areas, establishing the diagnosis remains challenging, and many cases are likely missed. However, because abscess formation (especially in the liver, spleen, and soft tissue, although prostatic abscesses were additionally reported in Northern Australia) is frequently observed in melioidosis and easily assessable by ultrasound,^7–9^ we hypothesized that POCUS might provide a valuable adjunct diagnostic tool to corroborate the diagnosis and allow early initiation of effective antimicrobial treatment. The latter is critical because melioidosis has an overall case fatality rate of up to 40%,^9^ and *B. pseudomallei* requires treatment with ceftazidime or a carbapenem, two drugs not often used as first-line empiric treatment of sepsis.^10^

This study on melioidosis was part of a larger observational diagnostic study, investigating the value of POCUS in the clinical management of febrile patients admitted to Mahosot Hospital, the primary reference hospital of Laos, in Vientiane. The study was prospectively performed in the hospital’s adult infectious diseases ward, enrolling consecutively admitted febrile patients between August 2016 and December 2016. Fever on admission was the sole inclusion criterion, and inability to undergo an ultrasound examination and unwillingness to participate in the study were the only exclusion criteria.

Patient data were collected on detailed case report forms and entered into an electronic database; the data were cleaned by checking for consistency and missing data. The POCUS examinations were performed using a portable black-and-white ultrasound machine (DP-30 with 3.5 MHz-convex and 7.5 MHz linear probe, Mindray, China) by a study clinician (K. K.) as soon as possible after hospital admission, usually on the day of admission. A description of the standard operating procedure for the POCUS examination is provided in Supplemental Appendix 1. In brief, an ultrasound examination assessing for pericardial and pleural effusions, ascites, abdominal lymphadenopathy, and abscesses in solid organs was performed using a convex probe. A linear probe was additionally used to assess the spleen for small abscesses and evaluate soft tissue abscesses when present. Sonographic findings were saved as digital pictures in patients with normal findings and as video clips in patients with abnormal findings. The Mahosot Hospital radiology department provided timely on-site expertise and served as a reference in the event of unclear sonographic findings. In addition, video clips of abnormal findings were reviewed externally (T. H.) to ensure quality control. Data were analyzed using descriptive statistics. For data analysis, we only used POCUS results of patients with a definite diagnosis, obtained using data from routine clinical care according to the locally available and established diagnostic standards, comparing patients with melioidosis and those with an alternative diagnosis. Confirmed melioidosis was defined by a positive *B. pseudomallei* culture result from any clinical sample (e.g., blood, urine, throat swab, sputum, and pus) conducted at the microbiology laboratory of Mahosot Hospital. *P*-values were calculated using the Mann–Whitney U-test for continuous variables and Fisher’s exact *t*-test for categorical variables. The study was approved by the National Ethics Committee for Health Research in Laos and by the Ethics Committee of Northwest and Central Switzerland. Written informed consent was obtained from all study participants or their legal guardians.

During the study period, 153 patients were included of whom 76 were men. In 111 patients, a definite diagnosis was obtained, including microbiologically confirmed melioidosis in 18 patients. Clinical characteristics and ultrasound findings are shown in Table 1. Patients with melioidosis were older than patients with an alternative diagnosis and were more likely to have underlying diabetes mellitus and less likely to be HIV positive. Pathological findings on ultrasound were generally common in patients both with and without melioidosis. However, the presence of abscesses was significantly more common in patients with melioidosis (Table 1, Figure 1). Eleven (61%) patients with melioidosis had an abscess at one or more sites compared with one (1%) patient with an alternative diagnosis (*P* < 0.0001). The positive and negative predictive value of finding one or more abscesses in patients with melioidosis in our patient population was 92% (60–99%) and 93% (88–96%), respectively.

**Table 1:** Point-of-care ultrasound findings in patients admitted with fever in Vientiane, Laos

	All patients (*n* = 153)	Patients with melioidosis (*n* = 18)	Patients with alternative diagnosis* (*n* = 93)	*P*-value†
Baseline characteristics				
Men (%)	76 (50)	11 (61)	42 (45)	0.14
Median age (years) (IQR)	30 (22–42)	53 (45–62)	29 (22–38)	< 0.0001
Diabetes	16 (10)	8 (44)	4 (4)	< 0.0001
HIV positive (%)	36 (24)	0 (0)	23 (25)	0.02
Ultrasound findings (%)				
Positive ultrasound findings	80 (52)	10 (56)	45 (48)	0.62
Pericardial effusion‡	20 (14)	1 (6)	11 (12)	0.69
Abdominal lymph nodes	6 (4)	0 (0)	4 (4)	1.0
Pleural effusion	50 (33)	5 (28)	23 (25)	0.77
Intra-abdominal effusion	25 (16)	0 (0)	9 (10)	0.35
Gall bladder edema	10 (7)	1 (6)	7 (8)	1.0
Liver abscess§	4 (3)	2 (11)	1 (1)‖	0.07
Spleen abscess§	7 (5)	4 (22)	0 (0)	0.0005
Prostate abscess§	3 (2)	1 (6)	0 (0)	0.16
Soft tissue abscess§	8 (5)	7 (39)	0 (0)	< 0.0001
Composite ultrasound findings (%)				
Any abscess¶	18 (12)	11 (61)	1 (1)¦	< 0.0001
Abscesses in multiple sites¶^,^#	4 (3)	3 (17)	0 (0)	0.004
Liver and/or splenic abscess	9 (6)	5 (28)	1 (1)¦	0.0004

* Most common alternative diagnoses included dengue (*n* = 47), rickettsiosis (*n* = 13), HIV-related opportunistic disease (*n* = 11), and urinary tract infection (*n* = 6).

† All *P*-values are provided for the comparison between patients with melioidosis and those with an alternative diagnosis. Some patients did not have a definite final diagnosis; they were not included in the analysis.

‡ All pericardial effusions observed in this study were relatively small, with a maximum diameter of 12 mm.

§ Hypoechoic lesions on ultrasound were interpreted as abscesses.

‖ ¦ This patient had a confirmed diagnosis of dengue fever. This diagnosis does not explain the presence of a liver abscess, but unfortunately invasive microbiological diagnostics to establish the cause of the liver abscess were unavailable.

¶ Including liver, splenic, prostatic, and soft tissue abscesses.

# Abscesses in multiple sites were only observed in patients with melioidosis and in one patient without a definitive diagnosis. In this patient, ultrasound demonstrated liver and splenic abscesses, suggesting melioidosis despite negative culture results.

**Figure 1.**
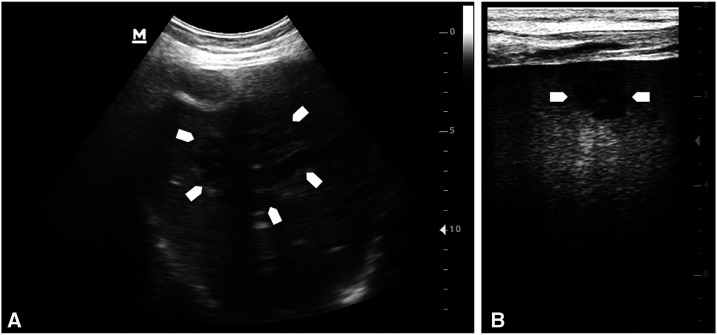
Hypoechoic lesions (arrows) due to melioidosis abscesses in the (**A**) liver (3.5 MHz convex) and (**B**) spleen (7.5 MHz linear probe). The hypoechoic lesions in the liver and spleen were frequently septated and/or localized in clusters which may suggest “honeycomb” or “necklace” appearance; because of the low image quality of the point-of-care ultrasound machine, these possibly diagnostic characteristics of the ultrasound images were not included in the analysis.

Our findings provide information on the diagnostic accuracy of positive ultrasound findings (abscesses) to diagnose melioidosis in patients admitted with fever in Laos and are in line with previous studies on ultrasound findings in patients with melioidosis in Southeast Asia. A retrospective study in patients with culture-proven melioidosis in India demonstrated that 23/189 (12%) had liver abscesses, a similar proportion to the 11% in our study.^11^ In Thailand, a prospective observational study in 230 patients with culture-confirmed melioidosis found one or more abscesses in the liver and/or spleen in 77 (33%) patients,^12^ similar to the 28% observed in our study. Other studies have demonstrated that the combination of liver and splenic abscesses can be predictive for melioidosis.^7,13^ In our study, the number of patients with multiple abscesses was low, but three of four patients with multiple abscesses were confirmed with melioidosis. In two patients with prostatic abscesses and three patients with splenic abscesses, including one patient who also had liver abscesses, no definite diagnosis was made. Possibly, these patients were true cases of melioidosis where a firm diagnosis might have been reached had it been possible to aspirate the abscesses. We found only one patient with a hypoechoic liver lesion who had a confirmed alternative diagnosis. This patient was found to have dengue fever. As dengue fever does not cause abscesses, another infection or a noninfectious pathology may have been present.

Four main limitations of our study need to be mentioned. First, the sample size was limited. Second, the diagnostic accuracy of ultrasound was calculated based on the analysis of culture confirmed cases of melioidosis. Because the sensitivity of culture is not perfect and some referred patients may have received antibiotic treatment before diagnostic blood sampling, some cases may have been missed. Third, our study was carried out during the dengue season. This may have increased our positive predictive value as a large proportion of our control group were patients with dengue infection. In other seasons, the proportion of febrile patients with alternative causes of abscesses such as amebic disease or staphylococcal infection may be higher. Finally, we used a low-cost ultrasound machine, which may have limited the detection of very small abscesses as well as the morphological characteristics suggestive of melioidosis (such as the “necklace” or “honeycomb” appearance previously described in melioidosis).^11^ Nevertheless, we intentionally opted for using simple equipment and simple image characteristics, as these more realistically reflect the conditions under which POCUS is applied. Furthermore, by adding a linear probe for screening of the spleen and soft tissue, we allowed for more sensitive detection of small abscess. However, because of the small sample size, it is difficult to ascertain whether morphological features are helpful in strengthening the tentative diagnosis of melioidosis in settings comparable with ours.

Although ultrasound cannot be used to rule out melioidosis, as not all patients with melioidosis develop abscesses, the presence of abscesses had a high positive predictive value in our study population. This cannot necessarily be extrapolated to other places and time periods, depending on the local epidemiology of other conditions such as amebic liver disease and staphylococcal infection. An additional differential diagnosis includes abscesses due to disseminated tuberculosis, although the patient populations at risk are significantly different. Whereas underlying diabetes and renal impairment may suggest melioidosis, HIV infection and malnutrition may indicate tuberculosis. Nevertheless, these differentials illustrate the limitations of imaging and the need for microbiological confirmation in equivocal cases. However, our data indicate that in a melioidosis-endemic area, the detection of abscesses in the liver, spleen, and soft tissue by bedside ultrasound has a high positive predictive value for melioidosis, especially when abscesses are detected in multiple sites in high-risk patients. In these patients, empiric antibiotic treatment covering *B. pseudomallei* should, thus, be initiated even in the absence of positive cultures.

## Supplemental appendix

Supplemental materials
