# Supplementary material for: Point-of-Care Ultrasound in the Diagnosis of Melioidosis in Laos
Source: Am J Trop Med Hyg. 2020 Jun 1;103(2):675–8. doi: 10.4269/ajtmh.20-0069 (PMC7410443; doi:10.4269/ajtmh.20-0069)
Supplement: Supplementary file 1 [file tpmd200069.SD1.docx]

**Supplemental Appendix 1: Standard operating procedure for Point-of-care ultrasound (POCUS)**

**1. Equipment:**

- Bed: For the ultrasound examination the patient has to be in a supine position and the examiner needs to be on the patient’s right side.
- Ultrasound machine model: a portable Mindray DP-20 will be used for all ultrasound investigations.
- Probes: Besides the probe position assessing the spleen, an electronic convex array transducer (35C50EB) will be used in all probe positions; in the probe position assessing the spleen an electronic linear array transducer (75L53EA) will be used. If soft-tissue abscesses are present the linear probe will be used to assess them.
- Ultrasound gel: Ultrasound gel is used as conductive medium between the patient’s skin and the ultrasound transducer and will be used in every ultrasound examination.

**2. Procedural Steps**

- All eligible patients fulfilling the study’s inclusion criteria will be invited to participate in the study.
- Eligible patients willing to participate will be enrolled after they have been informed on the study’s aim and after the ultrasound procedure has been explained.
- The lights in the room will be turned off and the room darkened, if possible.
- The patient will be placed in a supine position and the ultrasound machine set up on the right side of the patient.
- The patient’s study number will be registered in the ultrasound machine prior to starting the examination.
- A reasonable amount of ultrasound gel will be put on the probe.
- The bedside ultrasound examination will be started following the protocol specified below.

**3. Scanning positions**


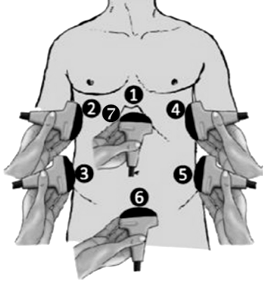


**Position 1A: pericardial effusion**

a. *Probe position:* The probe is placed transverse in the epigastric angle and the transducer is then tilted cranially to obtain a view on the heart. Asking the patient to inspire help to displace the heart caudally and improve visualization.

b. *Normal finding:* The pericardium is seen as an echogenic structure above the liver and diaphragm and below the heart; the parietal and visceral pericardium are inseparable.

c. *Pathological finding:* Pericardial effusion shows as an anechoic, black rim around the heart separating the parietal and visceral pericardium. Within the pericardial fluid echogenic material from inflammatory stranding may be seen. Isolated fluid at the apex is a normal finding and will not be reported as pericardial effusion.

d. *Interpretation:* An anechoic rim surrounding the heart confirms pericardial effusion and will be documented as such.

**Position 1B: abdominal lymphnodes**

a. *Probe position:* Four standard planes will be applied to visualize abdominal lymphnodes: 1) oblique porta hepatis, 2) transverse through head of pancreas, 3) splenic hilar, and 4) transverse mid abdomen.

b. *Normal finding:* Many physiological structures going beyond the scope of FASH can be seen.

c. *Pathological finding:* Lymphnodes appear as hypoechoic round or oval structures increasing in size as the transducer moves towards the centre of the node, and then diminishing again as the opposite side is reached. The nodes will be measured in their short axis. Central necrosis of lymphnodes may be seen.

d. *Interpretation:* In children, lymph nodes >10 mm (short axis) will be considered pathological and documented respectively. In adults, lymph nodes >15 mm (short axis) will be considered pathological and documented respectively. If present, central necrosis of lymphnodes will be documented.

**Position 2: pleural effusion right side**

a. *Probe position:* After the patient is asked to put his arms behind the neck, the transducer is positioned dorsal of the right mid-axillary line at the caudal part of the thorax with the long axis parallel to the ribs.

b. *Normal findings:* The diaphragm and the apical parts of the liver will be seen. Air in the basal parts of the lung will cause artefacts resembling a curtain that moves up and down with respiration.

c. *Pathological findings:* Anechoic black fluid may be seen in the costo-phrenic angle; within the pleural fluid echogenic material from inflammatory stranding may be seen. Hepatized lung tissue, the absence of A-lines and bronchograms may be seen.

d. *Interpretation:* Fluid in the costo-phrenic angle will be documented as pleural effusion. Hepatized lung tissue with absence of A-lines and possible concurrent bronchograms will be documented as lung consolidation.

**Position 3A: ascites in the hepato-renal pouch (Morrison’s Pouch)**

a. *Probe position:* The transducer is moved a few centimetres caudally and rotated so that the longitudinal axis is parallel to the body long axis.

b. *Normal finding:* The caudal edge of the liver and the kidney are visible with an echogenic white line between them.

c. *Pathological finding:* Anechoic fluid is visible between the liver and the kidney or around the kidney. Echogenic material from inflammatory processes may be floating within the fluid.

d. *Interpretation:* Presence of free fluid will be documented as ascites.

**Position 3B: right kidney**

a. *Probe position:* The probe is place in the right lower intercostal space in the midaxillary line and the liver may be used as “acoustic window”. After assessing the kidney in the longitudinal view (long axis) the probe is rotated and the kidney is scanned in the transverse (short axis) view to visualize the whole organ.

b. *Normal finding:* The kidney is invested in an echogenic capsule (Gerota’s fascia) surrounded by perinephritic fat, which in turn is surrounded by fascia. The kidney may be devided in renal cortex and medulla (parenchyma with a similar texture as liver tissue) which surrounds the echogenic renal sinus consisting of the pelvicalyceal system, renal vessels and fat. The calyces unite in the renal pelvis, which is the funnel-shaped origin of the ureter. The ureters are generally not well visualized, unless distended.

c. *Pathological finding:* Renal calculi / stones are brightly echogenic and demonstrate posterior acoustic shadowing. Dilatation of the renal pelvis and calyces (Hydronephrosis) points to an obstructed outflow of urine.

d. *Interpretation:* Presence of stones and hydronephrosis will be documented.

**Positions 3C: focal liver lesions**

**Position 3C-I: liver longitudinal**

a. *Probe position:* After the patient is asked to put his arms behind the neck, the probe is placed longitudinal in the epigastric angle and the transducer is then tilted to the left assessing the left liver lobe. Then the transducer is sliding to the right (finally ending at the right flank) trying to visualize as much liver tissue as possible. Asking the patient to inspire (if age appropriate) may help to displace the liver caudally and improve visualization.

b. *Normal finding:* The liver is seen as a moderately echogenic homogenous organ. Structures above the liver are the diaphragm and the heart; anechoic normal structures are intrahepatic vessels and the gallbladder.

c. *Pathological finding:* Focal non-linear hypoechoic lesions within the liver tissue (e.g. tuberculous granuloma or abscesses).

d. *Interpretation:* Focal non-linear hypoechoic lesions, possibly depicting tuberculous granuloma or abscesses, will be documented. In addition, the number, size, distribution and morphology of these lesions will be documented.

**Position 3C-II: liver transcostal**

a. *Probe position:* The transducer is moved slightly up and turned counter-clockwise until parallel to the ribs (intercostal window) to visualize the liver tissue. Similar images as in the positions 3b-I are achieved.

b. *Normal findings:* The liver is seen as a moderately echogenic homogenous organ and linear hypoechoic vessels are seen. The air above the pleura may be seen as echogenic artefact.

c. *Pathological finding:* Focal non-linear hypoechoic lesions within the liver tissue (e.g. tuberculous granuloma or abscesses).

d. *Interpretation:* Focal non-linear hypoechoic lesions, possibly depicting tuberculous granuloma or abscesses, will be documented. In addition, the number, size, distribution and morphology of these lesions will be documented.

**Position 3C-III: liver subcostal**

a. *Probe position:* The probe is placed transverse in the epigastric angle and the transducer is then tilted cranially to caudally to obtain a view on the liver. Asking the patient to inspire (if age appropriate) may help to displace the liver caudally and improve visualization.

b. *Normal finding:* The liver is seen as a moderately echogenic homogenous organ. Structures above the liver are the diaphragm and the heart; anechoic normal structures are intrahepatic vessels (hepatic veins and portal vein) and the gallbladder.

c. *Pathological finding:* Focal non-linear hypoechoic lesions within the liver tissue (e.g. tuberculous granuloma or abscesses).

d. *Interpretation:* Focal non-linear hypoechoic lesions, possibly depicting tuberculous granuloma or abscesses, will be documented. In addition, the number, size, distribution and morphology of these lesions will be documented.

**Position 4: pleural effusion left side**

a. *Probe position:* Mirroring position 2 the transducer is placed on the left side of the caudal thorax dorsal of the right mid-axillary line with the long axis parallel to the ribs.

b. *Normal findings:* The diaphragm and spleen will be seen and in the absence of pleural effusion air in the basal parts of the lung will cause artefacts resembling a curtain that moves up and down with respiration.

c. *Pathological findings:* Anechoic black fluid may be seen in the costo-phrenic angle; within the pleural fluid echogenic material from inflammatory stranding may be seen. Hepatized lung tissue, the absence of A-lines and bronchograms may be seen.

d. *Interpretation:* Fluid in the costo-phrenic angle will be documented as pleural effusion. Hepatized lung tissue with absence of A-lines and possible concurrent bronchograms will be documented as lung consolidation.

**Position 5A: focal splenic lesions**

a. *Probe position:* The transducer is moved slightly upwards and paralleled to the ribs to visualize the spleen.

b. *Normal findings:* Homogenous tissue of the spleen is visible.

c. *Pathological findings:* Hypoechoic dark lesions are visible within the splenic tissue.

d. *Interpretation:* Hypoechoic dark lesions, possibly depicting abscesses, will be documented.

**Position 5B: ascites in the spleno-renal pouch (Koller’s Pouch)**

a. *Probe position:* The transducer is moved caudally mirroring position 3a and visualizing spleen and left kidney.

b. *Normal findings:* Spleen and kidney appear separated by a white echogenic line representing the capsules of the organs.

c. *Pathological findings:* Analogous to the hepato-renal pouch free fluid will be seen as black anechoic fluid in the spleno-renal pouch.

d. *Interpretation:* Free fluid in the spleno-renal pouch will be documented as ascites.

**Position 5C: left kidney**

a. *Probe position:* The transducer positioned and moved mirroring position 3b.

b. *Normal finding:* see 3b.

c. *Pathological finding:* see 3b.

d. *Interpretation:* see 3b.

**Position 6A: ascites in the pouch of Douglas (female) or recto-vesical pouch (male)**

a. *Probe position:* The probe is placed on the lower abdomen touching the upper of the symphysis pubis. The pelvic region will be scanned in the longitudinal and transverse axis.

b. *Normal findings:* The bladder will be visible and varying in size depending urine content. In female patients the uterus may be visible as a “pear shaped” organ behind the bladder. In male patients the recto-vesical pouch is located between the rectum and the bladder.

c. *Pathological findings:* Echo-free black areas may be seen behind the bladder (male patients) or uterus (female patients) and represent free fluid in the recto-vesical and Douglas pouch, respectively.

d. *Interpretation:* Free fluid in the recto-vesical or Douglas pouch represents ascites and will be documented as such.

**Position 6B: focal prostatic lesions**

a. *Probe position:* The probe will be tilted to scan the prostate located caudally to the bladder.

b. *Normal findings:* Homogenous tissue of the round- to oval-shaped prostate is visible.

c. *Pathological findings:* Hypoechoic dark lesions visible within the prostatic tissue.

d. *Interpretation:* Hypoechoic dark lesions, possibly depicting abscesses, will be documented.

**Position 7: gall bladder**

a. *Probe position:* Identify the gall bladder by a longitudinal scan in the mid-axillary line at the costal margin (altering the probe angle to scan between the ribs may be helpful) or place the probe subcostal and sweep down and laterally.

b. *Normal finding:* The gall bladder appears as well-demarcated fluid-filled structure inferior to the liver. Normal gall bladder wall thickness is ≤3mm in patients who fasted.

c. *Pathological finding:* Calculi / stones are brightly echogenic and demonstrate posterior acoustic shadowing. A thickened wall often appears as two echogenic lines with a hypoechoic region between them. This suggests acute inflammation and/or oedema. Besides cholecystitis, oedema is also seen in sepsis and capillary leackage due to dengue. CAVE: The gall bladder wall will also be thickened due to contraction in non-fasted patients!

d. *Interpretation:* The presence of calculi / stones and the presence of a thickened gall bladder wall in patients who fasted will be documented.

**Soft-tissue abscesses**

In the case apparent soft-tissue abscess(es) are present their number, location and size will be assessed by ultrasound and recorded, respectively.

**4. Documentation**

**4.1 Document of POCUS results**

- The findings of the POCUS examination will be documented in the "Laos-POCUS-study - Sonographical CRF"
- The positive and negative findings will be documented for each probe position, respectively.
- In the case of parenchymal liver and spleen lesions, their number, distribution, size and morphology will be recorded. The distribution of lesions was classified as "discrete" for scattered lesions separated by intervening normal parenchyma and "clustered" for multiple lesions located close together.
- The lesions will be documented as "hypoechoic" for simple round, hypo- to anechoic lesions without internal structure, "target" for small, hypoechoic lesions with a tiny central echogenic spot, "bull’s eye" for lesions with a larger central echogenic area, "satellites" for lesions with multiple smaller surrounding lesions, "honey-comb" for lesions with internal septa or locules that might represent coalescence of multiple small lesions to form a large abscess and "necklace" for multiple peripheral radial loculations contained within the larger hypoechoic honeycomb lesions. Schematic drawings are given below. Comments are possible, including free-text descriptions of the lesions’ morphology in the CRF.
- The size of the lesions will be documented by using the digital measuring tool of the ultrasound machine.


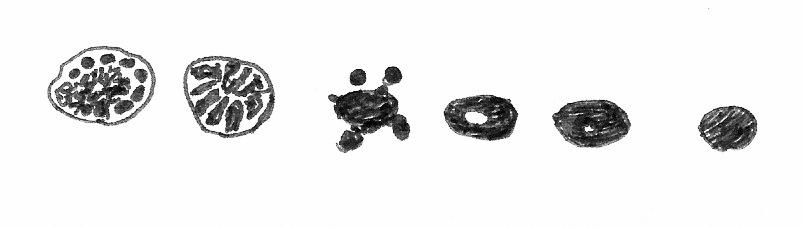


Figure 2. From left to right: "hypoechoic", "target", "bull’s eye", "satellite", "honey-comb", "necklace" shape of focal lesions in the liver

**4.2 Documentation of visualisation conditions**

The general visualisation conditions during the POCUS examination will be documented categorizing visualisation into "satisfactory", "partly obscured" and "obscured".

**4.3 Saving of ultrasound scans**

In every position of the POCUS examination, a scan picture will be electronically saved on the ultrasound machine’s internal harddisk to document normal as well as pathological findings. Pathological findings will additionally be saved as video clips on the ultrasound machine’s internal harddisk.
